# Relative leucocyte telomere length is associated with incident end-stage kidney disease and rapid decline of kidney function in type 2 diabetes: analysis from the Hong Kong Diabetes Register

**DOI:** 10.1007/s00125-021-05613-1

**Published:** 2021-11-22

**Authors:** Feifei Cheng, Andrea O. Luk, Hongjiang Wu, Claudia H. T. Tam, Cadmon K. P. Lim, Baoqi Fan, Guozhi Jiang, Luke Carroll, Aimin Yang, Eric S. H. Lau, Alex C. W. Ng, Heung Man Lee, Elaine Chow, Alice P. S. Kong, Anthony C. Keech, Mugdha V. Joglekar, Wing Yee So, Anandwardhan A. Hardikar, Juliana C. N. Chan, Alicia J. Jenkins, Ronald C. W. Ma

**Affiliations:** 1 Department of Medicine and Therapeutics, The Chinese University of Hong Kong, Prince of Wales Hospital, Hong Kong, SAR China; 2 Hong Kong Institute of Diabetes and Obesity, The Chinese University of Hong Kong, Prince of Wales Hospital, Hong Kong, SAR China; 3 Li Ka Shing Institute of Health Sciences, The Chinese University of Hong Kong, Prince of Wales Hospital, Hong Kong, SAR China; 4 NHMRC Clinical Trial Centre, Faculty of Medicine and Health, University of Sydney, Sydney, NSW Australia; 5 Diabetes and Islet Biology Group, School of Medicine, Western Sydney University, Sydney, NSW Australia; 6 The Chinese University of Hong Kong-Shanghai Jiao Tong University Joint Research Centre in Diabetes Genomics and Precision Medicine, Prince of Wales Hospital, Hong Kong, SAR China

**Keywords:** Chinese, End-stage kidney disease, Kidney function, Telomere length, Type 2 diabetes

## Abstract

**Aims/hypothesis:**

Few large-scale prospective studies have investigated associations between relative leucocyte telomere length (rLTL) and kidney dysfunction in individuals with type 2 diabetes. We examined relationships between rLTL and incident end-stage kidney disease (ESKD) and the slope of eGFR decline in Chinese individuals with type 2 diabetes.

**Methods:**

We studied 4085 Chinese individuals with type 2 diabetes observed between 1995 and 2007 in the Hong Kong Diabetes Register with stored baseline DNA and available follow-up data. rLTL was measured using quantitative PCR. ESKD was diagnosed based on the ICD-9 code and eGFR.

**Results:**

In this cohort (mean ± SD age 54.3 ± 12.6 years) followed up for 14.1 ± 5.3 years, 564 individuals developed incident ESKD and had shorter rLTL at baseline (4.2 ± 1.2 vs 4.7 ± 1.2, *p* < 0.001) than the non-progressors (*n* = 3521). On Cox regression analysis, each ∆∆C_t_ decrease in rLTL was associated with an increased risk of incident ESKD (HR 1.21 [95% CI 1.13, 1.30], *p* < 0.001); the association remained significant after adjusting for baseline age, sex, HbA_1c_, lipids, renal function and other risk factors (HR 1.11 [95% CI 1.03, 1.19], *p* = 0.007). Shorter rLTL at baseline was associated with rapid decline in eGFR (>4% per year) during follow-up (unadjusted OR 1.22 [95% CI 1.15, 1.30], *p* < 0.001; adjusted OR 1.09 [95% CI 1.01, 1.17], *p* = 0.024).

**Conclusions/interpretation:**

rLTL is independently associated with incident ESKD and rapid eGFR loss in individuals with type 2 diabetes. Telomere length may be a useful biomarker for the progression of kidney function and ESKD in type 2 diabetes.

**Graphical abstract:**

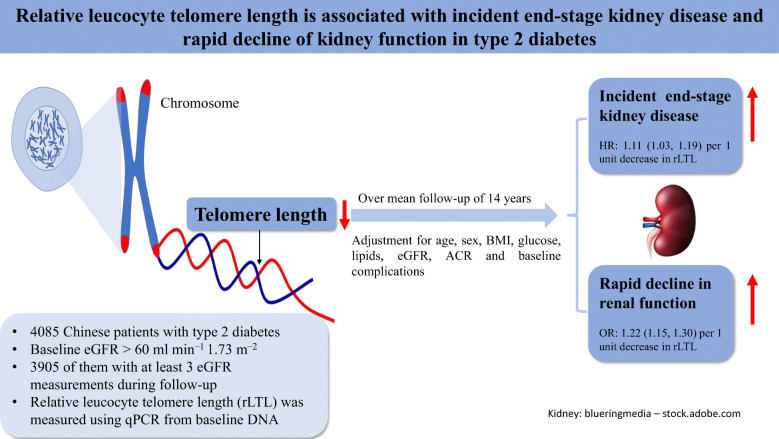

**Supplementary Information:**

The online version contains peer-reviewed but unedited supplementary material available at 10.1007/s00125-021-05613-1.

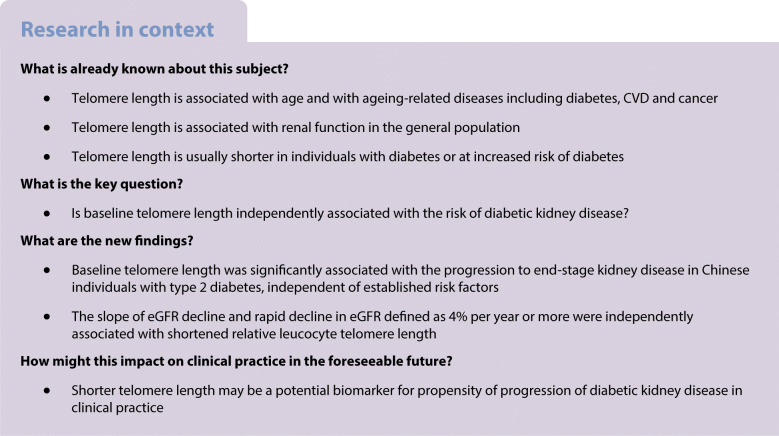



## Introduction

Diabetes, in particular type 2 diabetes, is the leading cause of end-stage kidney disease (ESKD), and this complication is highly prevalent in Asian populations [1]. In a population-based retrospective cohort analysis of 338,908 Chinese individuals with diabetes during 2000–2012, the crude incidence of ESKD was 11.3 per 1000 person-years [2]. The rate of disease progression varied among patients with type 2 diabetes and was subject to influence by metabolic control and genetic factors [3]. There are global programmes for biomarker discovery aimed at identifying individuals with type 2 diabetes at high risk of developing ESKD or rapid decline in kidney function for timely intervention.

Telomeres are tandem repeats of 5′-TTAGGG-3′ sequences at the ends of each DNA strand within every chromosome [4]. Telomere length is a useful biomarker of chronological age and age-related disorders such as CVD [5], mortality [6] and cancer [7]. In a cross-sectional study, 137 patients with ESKD had shorter leucocyte telomere length (LTL) than 144 healthy controls [8, 9]. Several reports in cross-sectional cohorts had demonstrated shorter LTL in individuals with type 2 diabetes and microalbuminuria vs those without [4, 10, 11]. In both the general population [12] and individuals with type 2 diabetes [5], LTL was cross-sectionally associated with eGFR and urinary albumin/creatinine ratio (ACR). In a 3 year prospective study of 691 Asian individuals with type 2 diabetes, there was an inverse association between shorter LTL and increased risk of progression of albuminuria [13].

Although these findings suggested that shorter LTL might be associated with increased risk of kidney dysfunction, their significance was limited by small sample size, cross-sectional observation or short follow-up period. In this study, we examined the relationships between LTL and incident ESKD in a large prospective cohort of Chinese individuals with type 2 diabetes. Due to the long follow-up period, we also investigated the relationships between baseline LTL and slope of decline of eGFR and rapid loss of kidney function.

## Methods

### Study population

A total of 5506 consecutively recruited adult individuals with type 2 diabetes and who had available DNA and clinical data were selected from the Hong Kong Diabetes Register (HKDR) between 1995 and 2007 [14]. The details of enrolment and assessment has been described elsewhere [14, 15]. Established since 1995, the HKDR enrolled Chinese individuals with diabetes attending the Prince of Wales Hospital (PWH) and other community clinics as a research-driven quality improvement programme. All participants were referred to the PWH Diabetes Mellitus and Endocrine Centre for comprehensive assessment of metabolic control and complications based on the European DIABCARE protocol after an overnight fast [16, 17]. Upon enrolment, all participants provided written informed consent for donating additional blood and urine collection for research purposes. This study was approved by the Joint Chinese University of Hong Kong - New Territories East Cluster Clinical Research Ethics Committee.

### Relative LTL measurements

DNA was extracted from the whole blood of participants and stored in freezers after extraction [5]. DNA extractions were mainly conducted from 1997 onwards, with most samples extracted within 1 year of blood collection, using a traditional phenol–chloroform method [5]. Telomere length measurements were conducted using an updated quantitative real-time PCR method [5, 18, 19] and were reported as ∆∆C_t_ between telomere and single-copy gene (encoding human β-globin [HBG]) relative to a normalisation control. A no-template control (NTC, water) and a reference human DNA sample for quality control (QC) were included for normalisation of any plate-to-plate variability and calculation of ∆∆C_t_. There is no consensus or agreed procedures for normalising relative LTL (rLTL) measurements, with calculations based on NTC or QC being acceptable in most studies. We compared the results derived from these two methods of normalisation to evaluate their consistencies [5, 6]. Samples with CV >2.5% were repeated and the inter-plate CVs of the telomere and HBG assays were 2.9% and 1.2%, respectively. The overall intra-plate CV was 1.2% for rLTL and 0.4% for HBG. We excluded 157 (2.9%) participants due to failed QC (*n* = 126) or missing rLTL measurement (*n* = 31).

### Definition of outcomes

Of the remaining 5349 participants, we excluded 1264 with chronic kidney disease (CKD) defined as eGFR <60 ml min^−1^ [1.73 m]^−2^ at baseline and thus included 4085 participants in the final analysis. Young-onset diabetes was defined as age of diagnosis <40 years. CKD was defined as follows: (1) fatal and non-fatal diabetes with renal manifestations (ICD-9 [http://www.icd9data.com/2007/Volume1/default.htm] code 250.4), CKD (ICD-9 code 585) or unspecified renal failure (ICD-9 code 586); (2) dialysis (ICD-9 procedure code 39.95) or peritoneal dialysis (ICD-9 procedure code 54.98); or (3) eGFR <60 ml min^−1^ [1.73 m]^−2^. A random urine sample was collected to define microalbuminuria (urinary ACR 2.5–30 mg/mmol in male participants or 3.5–30 mg/mmol in female participants) or macroalbuminuria (ACR >30 mg/mmol) [20]. Retinopathy was defined by the presence of dot-blot haemorrhages, hard exudates, cotton wool spots, neovascularisation, retinal laser scars or a history of vitrectomy [20]. ESKD was defined by the codes of dialysis (ICD-9 procedure codes 39.95 or 54.98), kidney transplant (ICD-9 procedure code 55.6 or diagnosis codes 996.81 or V42.0), or eGFR <15 ml min^−1^ [1.73 m]^−2^. The time to onset of ESKD was defined as the period from baseline visit to the date of incident ESKD or the censored date (30 June 2017), whichever came first.

A total of 3905 participants with at least three eGFR measurements during follow-up were included for calculation of the slope of eGFR. Linear mixed-effects regression was used to calculate eGFR slope for each individual and then re-expressed as the percentage change per year in eGFR. Rapid loss of kidney function was defined as >4% decline in eGFR per year [21–23].

As a sensitivity analysis, we further excluded ESKD that occurred following episodes of marked deterioration in renal function in a short timeframe. We defined acute kidney injury (AKI) according to the Kidney Disease Improving Global Outcomes (KDIGO) Acute Kidney Injury Work Group guidelines [24]. A total of 153 participants who had ESKD endpoint within 30 days of an AKI event were further excluded in the sensitivity analysis.

### Statistical analysis

All data are expressed as mean ± SD, median (Q1–Q3) or percentage (%), as appropriate. rLTL with skewness of −0.4 followed a normal distribution. Covariates with a skewed distribution were logarithmically transformed. We used Student’s *t* test, χ^2^ test or Fisher’s exact test for comparisons between groups, as appropriate. A general linear model was conducted to compare rLTL between groups after adjusting for other confounders. Multivariable Cox and logistic regression were performed to examine associations between baseline rLTL and outcomes. Linear regression was conducted to estimate the relationship between rLTL and the slope of eGFR decline. Traditional clinical risk factors for CKD in type 2 diabetes were selected from our previous study [25]. Since the proportional hazard assumptions for sex and retinopathy were not met, both sex and retinopathy at baseline were included as strata variables in Cox regression models, whereas other covariates were considered to have the same effects across strata. A total of 309 participants were excluded from the final model due to missing data. LDL-cholesterol and ACR had the largest number of missing data, accounting for 163 (4.0%) and 136 (3.3%) participants, respectively, while data for other variables were missing for fewer than 1% of participants. Given the low proportion of missing data, we did not undertake imputation of individual covariates in our analysis. As the association between rLTL and kidney outcomes was linear, we investigated rLTL as a continuous variable in regression analyses. The HR or OR with 95% CIs, represented the relative increase in the risk of outcomes associated with each ∆∆C_t_ decrease of rLTL. Besides, the Fine–Gray competing risk regression models were used to estimate the subdistribution HR of rLTL for incident ESKD, with death caused by other diseases entered as the competing risk. The association between rLTL (by tertile) and ESKD was assessed using the Kaplan–Meier method. A receiver operating characteristic (ROC) curve and a net reclassification improvement (NRI) were conducted to evaluate the contribution of rLTL as a predictor of ESKD. The likelihood ratio test and NRI were conducted to estimate whether rLTL had incremental value to the prediction models. NRI was calculated by using an R Package ‘PredictABEL’. All analyses were performed using R version 3.6.1 (www.r-project.org). A two-tailed *p* < 0.05 was considered statistically significant.

## Results

### Baseline characteristics of study participants

The 4085 participants had mean ± SD age of 54.3 ± 12.6 years, 45.4% were male, and participants had a mean diabetes duration of 6.0 ± 6.1 years (Table 1). During a mean ± SD follow-up period of 14.1 ± 5.3 years, 564 (13.8%) participants progressed to ESKD, with an incidence of 9.8 (95% CI 9.0, 10.7) per 1000 person-years. Among the 564 progressors, the mean ± SD diabetes duration was 8.0 ± 6.6 years at baseline, and the mean time to incident ESKD was 11.1 ± 5.1 years.

**Table 1:** Baseline characteristics of participants according to different kidney failure status during follow-up

Variable	Whole cohort	Non-progressors	Progressors	*p* value (non-progressors vs progressors)	*p* value after adjusting for age and sex
*N*	4085	3521	564		
Age, year	54.3 ± 12.6	53.7 ± 12.6	58.0 ± 12.3	<0.001	
Male, %	45.4	45.2	46.6	0.544	
Age at diagnosis, years	48.2 ± 12.3	48.0 ± 12.3	50.0 ± 12.7	<0.001	<0.001
Young age at diagnosis (<40 years), %	75.1	74.1	81.0	0.001	0.017
Duration of diabetes, years	6.0 ± 6.1	5.7 ± 5.9	8.0 ± 6.6	<0.001	<0.001
Current smoker, %	14.7	14.2	17.4	0.016	0.032
Ever smoked, %	29.4	28.7	33.7	0.054	0.024
Current drinker, %	10.2	10.5	8.2	0.115	0.107
Ever drinker, %	21.2	20.9	22.9	0.324	0.530
SBP, mmHg	132.3 ± 19.3	131.1 ± 18.9	140.1 ± 20.2	<0.001	<0.001
DBP, mmHg	76.0 ± 10.6	75.7 ± 10.4	77.6 ± 11.2	<0.001	<0.001
BMI, kg/m^2^	25.3 ± 4.1	25.3 ± 4.1	25.3 ± 4.1	0.962	0.341
HbA_1c_, mmol	59.7 ± 19.7	58.4 ± 18.8	67.5 ± 23.4	<0.001	<0.001
HbA_1c_, %	7.6 ± 1.8	7.5 ± 1.7	8.3 ± 2.1	<0.001	<0.001
FPG, mmol/l	8.6 ± 3.3	8.5 ± 3.1	9.6 ± 4.1	<0.001	<0.001
Total cholesterol, mmol/l	5.2 ± 1.1	5.1 ± 1.1	5.4 ± 1.2	<0.001	<0.001
HDL-C, mmol/l	1.3 ± 0.4	1.3 ± 0.4	1.3 ± 0.4	0.253	0.050
Non-HDL, mmol/l	3.8 ± 1.1	3.8 ± 1.1	4.0 ± 1.2	<0.001	<0.001
LDL-C, mmol/l	3.1 ± 1.0	3.1 ± 0.9	3.2 ± 1.0	<0.001	<0.001
Triacylglycerol, mmol/l	1.3 (0.9–2.0)	1.3 (0.9–1.9)	1.5 (1.0–2.1)	<0.001^a^	<0.001^a^
Urinary ACR, mg/mmol	1.6 (0.7–5.6)	1.4 (0.7–4.1)	7.5 (1.9–44.5)	<0.001^a^	<0.001^a^
eGFR, ml min^−1^ [1.73 m]^−2^	90.7 ± 16.8	91.8 ± 16.5	84.0 ± 17.6	<0.001	<0.001
Erythrocyte count, ×10^12^/l	4.7 ± 0.6	4.7 ± 0.6	4.6 ± 0.6	0.001	0.012
Haemoglobin, g/l	13.9 ± 1.5	14.0 ± 1.5	13.7 ± 1.5	0.001	0.001
WBC, ×10^9^/l	7.2 ± 2.8	7.2 ± 2.9	7.5 ± 2.0	0.003	0.010
Diagnosed comorbidity					
Retinopathy, %	23.0	20.1	41.7	<0.001	<0.001
Neuropathy, %	16.7	14.7	29.1	<0.001	<0.001
Microalbuminuria, %	25.8	24.2	35.5	<0.001	<0.001
Macroalbuminuria, %	9.8	6.5	30.9	<0.001	<0.001
Use of medications					
Lipid-lowering drugs, %	15.4	15.7	13.7	0.240	0.029
Antihypertensive drugs, %	38.6	36.5	51.8	<0.001	<0.001
Oral glucose-lowering drugs, %	67.4	66.8	70.7	0.073	0.415
Insulin, %	13.1	11.9	20.6	<0.001	<0.001
RAS inhibitors (ACEIs or ARBs), %	17.1	15.3	28.5	<0.001	<0.001
rLTL_NTC, ∆∆C_t_	4.6 ± 1.2	4.7 ± 1.2	4.2 ± 1.2	<0.001	<0.001
rLTL_QC, ∆∆C_t_	−0.1 ± 1.0	0.0 ± 1.0	−0.4 ± 1.1	<0.001	<0.001

Data are expressed as mean ± SD, median (Q1–Q3) or as a proportion (%)

All comparisons were adjusted for the differences of age and sex by using either general linear model for continuous data or logistic regression model for categorical data

^a^Logarithmic transformation was used in triacylglycerols and ACR

ACEI, angiotensin-converting enzyme inhibitor; ARB, angiotensin receptor blocker; DBP, diastolic BP; FPG, fasting plasma glucose; HDL-C, HDL-cholesterol; LDL-C, LDL-cholesterol; LTL_NTC, rLTL calculated by water; Non-HDL, non-HDL-cholesterol; RAS, renin–angiotensin system; SBP, systolic BP; WBC, white blood cell count

Compared with the participants who were free from ESKD, those with ESKD were older and had longer duration of diabetes at baseline. After adjusting for age and sex, ESKD progressors were more likely to be smokers and had higher BP, HbA_1c_ and worse lipid profiles than non-progressors. They also had higher urinary ACR and lower eGFR than non-progressors and were more likely to have retinopathy, sensory neuropathy, microalbuminuria and macroalbuminuria at baseline (Table 1).

### Risk association of baseline rLTL with incident ESKD

Progressors to ESKD had shorter rLTL than non-progressors and this difference remained significant after adjusting for age, sex, HbA_1c_, smoking status and albuminuria at baseline. Baseline characteristics according to tertiles of rLTL are presented in electronic supplementary material (ESM) Table 1. There was no difference in rLTL between men and women (4.5 ± 1.2 vs 4.6 ± 1.2, *p* = 0.283). We evaluated the independent risk association of shortened rLTL with incident ESKD in five models (Table 2). In the unadjusted model, one ∆∆C_t_ decrease in rLTL was associated with an HR of 1.21 (95% CI 1.13, 1.30; *p* < 0.001) for incident ESKD and this was attenuated to 1.15 (95% CI 1.08, 1.23; *p* < 0.001) after age and sex adjustment. After adjusting for traditional risk factors for CKD in type 2 diabetes (Model 5 in Table 2), the association between rLTL and incident ESKD remained significant (HR 1.11 [95% CI 1.03, 1.19]; *p* = 0.007). We also explored the relationship between rLTL and incident ESKD in male participants (*n* = 1853) and female participants (*n* = 2232) separately. In unadjusted models, shorter rLTL was associated with increased risk of ESKD in the same direction in both sexes. The risk association remained significant in female participants but not in male participants after adjusting for other risk factors. When participants were stratified by age at diabetes diagnosis, there were inverse associations between rLTL and incident ESKD in those with young-onset (<40 years of age) and late-onset (≥40 years of age) diabetes with or without adjustment (ESM Table 2). Results from the competing risk regression models were similar to those in the Cox regression models indicating no significant competing risk from death due to other diseases (ESM Table 3).

**Table 2:** Cox regression showing the association between rLTL and incident kidney failure

Variable	Model 1^a^		Model 2^b^		Model 3^c^		Model 4^d^		Model 5^e^	
	HR (95% CI)	*p* value	HR (95% CI)	*p* value	HR (95% CI)	*p* value	HR (95% CI)	*p* value	HR (95% CI)	*p* value
rLTL, ∆∆C_t_^f^	1.21 (1.13, 1.30)	<0.001	1.15 (1.08, 1.23)	<0.001	1.12 (1.05, 1.20)	0.001	1.11 (1.03, 1.19)	0.006	1.11 (1.03, 1.19)	0.007
Age, years			1.04 (1.03, 1.05)	<0.001	1.02 (1.01, 1.03)	<0.001	1.00 (0.99, 1.02)	0.470	1.01 (1.00, 1.02)	0.316
Diabetes duration, years					1.04 (1.03, 1.05)	<0.001	1.01 (1.00, 1.03)	0.125	1.00 (0.99, 1.02)	0.125
BMI, kg/m^2^					1.01 (0.99, 1.04)	0.171	0.99 (0.97, 1.01)	0.307	0.99 (0.97, 1.01)	0.433
SBP, mmHg					1.02 (1.02, 1.03)	<0.001	1.01 (1.01, 1.02)	<0.001	1.01 (1.01, 1.02)	<0.001
DBP, mmHg					0.98 (0.97, 0.99)	0.001	0.98 (0.97, 0.99)	<0.001	0.98 (0.97, 0.99)	<0.001
HbA_1c_, mmol/mol							1.01 (1.01, 1.02)	<0.001	1.01 (1.01, 1.02)	<0.001
LDL-C, mmol/l							0.86 (0.78, 0.95)	0.002	0.85 (0.77, 0.93)	0.001
Log_10_ triacylglycerol							1.38 (0.89, 2.14)	0.147	1.50 (0.96, 2.33)	0.073
HDL-C, mmol/l							1.21 (0.95, 1.54)	0.133	1.22 (0.96, 1.56)	0.102
eGFR, ml min^−1^ [1.73 m]^−2^							0.98 (0.97, 0.98)	<0.001	0.98 (0.97, 0.98)	<0.001
Log_10_ ACR							3.20 (2.80, 3.66)	<0.001	2.92 (2.54, 3.36)	<0.001
Neuropathy									1.20 (0.97, 1.47)	0.089
CVD at baseline									0.88 (0.67, 1.16)	0.356

Sex and retinopathy were included as strata variables

^a^Model 1: without adjustment

^b^Model 2: adjusted for age and sex

^c^Model 3: Model 2 + adjusted for duration of diabetes, BMI, SBP and DBP

^d^Model 4: Model 3 + adjusted for HbA_1c,_ LDL-C, log_10_ triacylglycerol, HDL-C, eGFR and log_10_ ACR

^e^Model 5: Model 4 + retinopathy, neuropathy and CVD at baseline

^f^∆∆Ct refers to each ∆∆Ct decrease in rLTL

DBP, diastolic BP; HDL-C, HDL-cholesterol; LDL-C, LDL-cholesterol; rLTL, relative LTL calculated by negative control (water); SBP, systolic BP

**Fig. 1:**
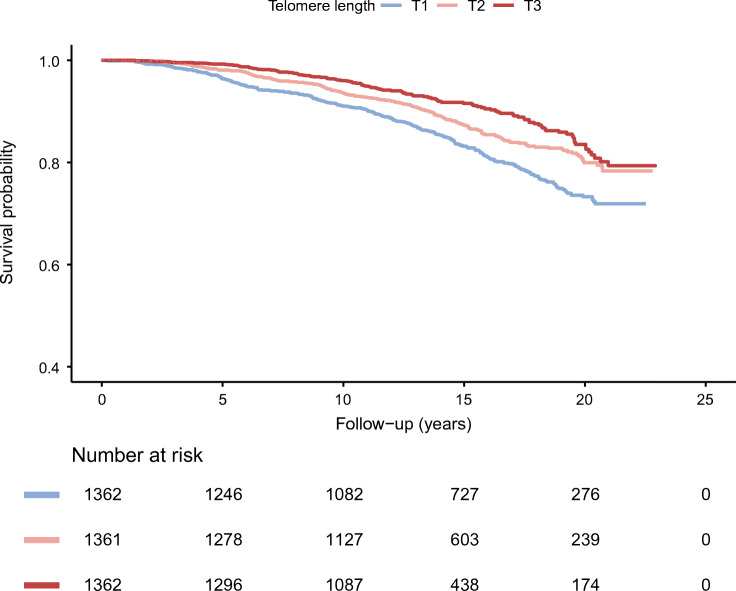
Cumulative survival probability of participants without new-onset kidney failure according to tertiles (T) of relative telomere length. *p* < 0.001 from the comparison across groups calculated by logrank test. T1, rLTL ≤ 4.20; T2, 4.20 < rLTL ≤ 5.13; T3, rLTL > 5.13

On Kaplan–Meier analysis, tertiles of rLTL (T1, rLTL ≤4.20; T2, 4.20 < rLTL≤5.13; T3, rLTL >5.13) exhibited clear separation for progression to incident ESKD (Fig. 1, *p* < 0.001, logrank test). Participants with the shortest rLTL (T1) had an HR of 1.77 (95% CI 1.43, 2.19; *p* < 0.001) for progressing to ESKD during follow-up when compared with participants with the longest rLTL (T3) (ESM Table 4). Adjustment for age, diabetes duration, BP, glucose and other traditional risk factors attenuated the association, which remained significant (HR 1.39 [95% CI 1.10, 1.75], *p* = 0.006; ESM Table 4).

In the ROC analysis, AUC was used to assess the discriminatory power of the model including rLTL in addition to traditional risk factors (fully adjusted model). The AUC was 0.785 (95% CI 0.764, 0.806) for the model including clinical variables alone and increased to 0.792 (95% CI 0.772, 0.813) when both clinical variables and rLTL were included (*p* = 0.002, ESM Fig. 1). An analysis using NRI yielded similar conclusions, with an estimated NRI of 4.8% (95% CI 0.9, 8.7, *p* = 0.016) after including rLTL.

### Risk association of rLTL and decline of eGFR

ESM Fig. 2 shows the distribution of eGFR change per year during follow-up. Among 3905 participants with at least three eGFR measurements during follow-up, 1057 (27.1%) were defined as rapid decliners (eGFR decline >4.0% per year) compared with the median change of −1.6% (IQR –4.3, −0.4) per year. The mean eGFR change per year was −2.4% in participants with the shortest rLTL (T1), compared with −1.5% in the group with the longest rLTL (T3).

Linear regression analysis showed a similar relationship between rLTL and eGFR slope analysed as continuous traits (ESM Fig. 3 and ESM Table 5). In the unadjusted model, baseline rLTL was positively associated with subsequent eGFR change per year with a β ± SE value of 0.42 ± 0.07 (*p*< 0.001). This was attenuated to 0.13 ± 0.06 in the fully adjusted model. We used the cut-off value of 4% per year to define rapid decline in eGFR. In the logistic regression, baseline rLTL was associated with an OR of 1.22 (95% CI 1.15, 1.30; *p* < 0.001) in the unadjusted model; the OR was attenuated but remained significant after adjustment for other covariates, with an OR of 1.09 (95% CI 1.01, 1.17; *p* = 0.024) (Table 3).

**Table 3:** The relationship between rLTL and rapid decline in renal function

Variable	Model 1^a^		Model 2^b^		Model 3^c^		Model 4^d^		Model 5^e^	
	OR (95% CI)	*p* value	OR (95% CI)	*p* value	OR (95% CI)	*p* value	OR (95% CI)	*p* value	OR (95% CI)	*p* value
Intercept	0.93 (0.71, 1.23)	0.615	0.07 (0.04, 0.11)	<0.001	0.01 (0.00, 0.02)	<0.001	0.03 (0.01, 0.15)	<0.001	0.03 (0.01, 0.15)	<0.001
rLTL, ∆∆C_t_^f^	1.22 (1.15, 1.30)	<0.001	1.18 (1.11, 1.25)	<0.001	1.15 (1.08, 1.22)	<0.001	1.09 (1.02, 1.17)	0.017	1.09 (1.01, 1.17)	0.024
Age, years			1.05 (1.04, 1.05)	<0.001	1.03 (1.02, 1.04)	<0.001	1.02 (1.01, 1.04)	<0.001	1.02 (1.01, 1.03)	<0.001
Male sex			1.07 (0.92, 1.24)	0.37	1.18 (1.02, 1.38)	0.032	1.08 (0.90, 1.29)	0.413	1.07 (0.89, 1.28)	0.466
Diabetes duration, years					1.04 (1.03, 1.05)	<0.001	1.01 (1.00, 1.03)	0.066	1.01 (0.99, 1.02)	0.347
BMI, kg/m^2^					1.01 (0.99, 1.03)	0.244	0.99 (0.97, 1.02)	0.589	1.00 (0.97, 1.02)	0.700
SBP, mmHg					1.02 (1.02, 1.03)	<0.001	1.02 (1.01, 1.02)	<0.001	1.02 (1.01, 1.02)	<0.001
DBP, mmHg					0.99 (0.98, 1.00)	0.092	0.98 (0.97, 0.99)	0.003	0.98 (0.97, 0.99)	0.004
HbA_1c_, %							1.20 (1.14, 1.26)	<0.001	1.18 (1.13, 1.24)	<0.001
LDL-C, mmol/l							1.00 (0.91, 1.09)	0.998	1.00 (0.91, 1.09)	0.919
Log_10_ triacylglycerol							1.23 (0.81, 1.87)	0.332	1.33 (0.87, 2.02)	0.191
HDL-C, mmol/l							0.78 (0.60, 0.99)	0.045	0.79 (0.61, 1.01)	0.059
eGFR, ml min^−1^ [1.73 m]^−2^							0.99 (0.98, 0.99)	<0.001	0.99 (0.98, 1.00)	<0.001
Log_10_ ACR							3.99 (3.39, 4.71)	<0.001	3.79 (3.21, 4.48)	<0.001
Retinopathy									1.35 (1.11, 1.64)	0.003
Neuropathy									1.38 (1.11, 1.71)	0.004
CVD at baseline									0.93 (0.71, 1.20)	0.571

Rapid renal function decline was defined as >4% decline in eGFR per year

^a^Model 1: without adjustment

^b^Model 2: adjusted for age and sex

^c^Model 3: Model 2 + adjusted for duration of diabetes, BMI, SBP and DBP

^d^Model 4: Model 3 + adjusted for HbA_1c,_ LDL-C, log_10_ triacylglycerol, HDL-C, eGFR and log_10_ ACR

^e^Model 5: Model 4 + retinopathy, neuropathy and CVD at baseline

^f^∆∆Ct refers to each ∆∆Ct decrease in rLTL

DBP, diastolic BP; HDL-C, HDL-cholesterol; LDL-C, LDL-cholesterol; rLTL, relative LTL calculated by negative control (water); SBP, systolic BP

### Sensitivity analyses

Several sensitivity analyses were performed to assess the robustness of these findings. First, we repeated the analysis by using incident CKD as outcome. Among 4085 participants, 2292 (56.1%) progressed to CKD during a mean follow-up of 10.3 years. Baseline rLTL was associated with incident CKD in the unadjusted model, with an HR of 1.12 (95% CI 1.08, 1.16; *p* < 0.001); the HR was attenuated to 1.04 (95% CI 1.00, 1.08; *p* = 0.032) after adjustment for age and sex. The relationship was rendered non-significant after adjustments for BP, diabetes duration and BMI (HR 1.02 [95% CI 0.99, 1.06], *p* = 0.196) and other risk factors (HR 1.00 [95% CI 0.96, 1.03], *p* = 0.777).

Second, since macroalbuminuria is a strong predictor for kidney dysfunction, we excluded 524 (12.8%) participants who had macroalbuminuria at baseline and eGFR >60 ml min^−1^ [1.73 m]^−2^. In the remaining 3561 patients who were free of CKD and macroalbuminuria at baseline (mean age 54.1 years, 45.1% male sex, mean diabetes duration 5.8 years), 376 (10.6%) developed ESKD during a mean follow-up period of 14.3 years. Cox regression analysis adjusted for traditional risk factors indicated that rLTL was associated with incident ESKD in these participants, with an HR of 1.20 (95% CI 1.10, 1.30; *p* < 0.001) in the unadjusted model; after adjusting for all confounders, the HR was attenuated to 1.08 (95% CI 0.99, 1.17; *p* = 0.092).

Third, we excluded 153 incident cases of ESKD that occurred within 30 days of the AKI event. The relationship between rLTL and incident ESKD remained and was just short of statistical significance in the fully adjusted model (ESM Table 6).

Finally, we evaluated the association between rLTL, calculated by the reference QC sample (rLTL_QC), and the risk of incident ESKD. Compared with non-progressors, the progressors had shorter rLTL_QC (−0.4 ± 1.1 vs 0.0 ± 1.0, *p* < 0.001; Table 1) and this remained significant after adjusting for age, sex, glucose control, smoking status and albuminuria (*p* < 0.001). Similar to the results of rLTL calculated using water, per ∆∆C_t_ decrease of baseline rLTL_QC was associated with an HR of 1.24 (95% CI 1.16, 1.34; *p* < 0.001) for incident ESKD during the follow-up period; this association did not change after sequential adjustments for traditional risk factors (HR 1.14 [95% CI 1.05, 1.23], *p* = 0.002) (ESM Table 7).

## Discussion

In this large prospective study of 4085 Chinese individuals with type 2 diabetes, we demonstrated independent risk associations between baseline rLTL and incident ESKD, CKD and eGFR decline. Both progression to ESKD and rapid decline in eGFR (defined as 4% per year or more) were independently associated with shorter baseline rLTL. When analysed as continuous traits, there were inverse and linear relationships between eGFR decline and baseline rLTL independent of traditional risk factors. Our detailed analysis included NRI and ROC analysis and supported the added value of rLTL on top of traditional risk factors in identifying individuals with type 2 diabetes at high risk of ESKD and rapid deterioration of kidney function for individualised and intensified treatment. The association between rLTL and eGFR decline also provided new insights regarding the biological significance of telomeres in the pathogenesis of kidney dysfunction in type 2 diabetes.

To our knowledge, this is the largest prospective study to comprehensively evaluate the relationship between baseline rLTL and the progression to CKD and ESKD in individuals with type 2 diabetes. In this study, the rate of eGFR decline was linearly associated with baseline rLTL and each ∆∆C_t_ decrease of rLTL was significantly associated with a 0.4% decline per year in eGFR. Each unit decline of rLTL was associated with a 1.22-fold increased risk of accelerated decline in kidney function defined as more than 4% decline in eGFR per year. These prospective data concorded with findings from previous small-scale or cross-sectional studies. Several studies had reported associations between shorter LTL at baseline and increased risk for progression of albuminuria [13, 26]. In the United States National Health and Nutrition Examination Survey, LTL was cross-sectionally associated with urinary ACR and eGFR in 10,568 participants [12]. However, these positive results are not always consistent. In a study from Japan, researchers reported associations between LTL and eGFR [27] while other studies did not find any significant association between LTL and kidney function [13, 28]. In a study including 157 individuals with type 1 diabetes and kidney disease and 116 individuals with normoalbuminuria followed for a mean of 11.1 years, LTL was not related to eGFR decline rate calculated by linear regression of serial eGFR measurements [29].

In a study including 889 non-dialysis-dependent individuals with a mean eGFR of 34 ml min^−1^ [1.73 m]^−2^, 293 (33%) had type 1 or type 2 diabetes [30]. The eGFR change was calculated based on two measurements expressed as difference in eGFR between baseline and last follow-up visit divided by the number of years without estimation of the slope or trajectory for eGFR decline [30]. Using these estimates, the researchers did not find any difference in LTL between tertiles of eGFR change in individuals with or without diabetes [30]. However, using the endpoint of doubling of baseline serum creatinine and/or incident ESKD [30], the researchers reported an association of shorter LTL with CKD progression only among the individuals with diabetes [30]. Of note, only 293 individuals had diabetes and 164 developed incident endpoints during a median follow-up period of 2.8 years. To this end, the robust findings from our cohort, with larger numbers and longer follow-up time with at least three eGFR measurements to estimate eGFR slope, lent support to the potential utility of using shorter rLTL as a biomarker for rapid loss of kidney function.

In our analysis, a one-unit decrease in rLTL was associated with 1.21-fold increased risk of incident ESKD, and this association was independent of traditional risk factors including age, duration of diabetes, metabolic control and complications at baseline. Compared with participants with the longest rLTL, those with the shortest rLTL had a 1.8-fold increased risk of ESKD. Previous prospective studies focused on progression of albuminuria in people with diabetes. In a study including 132 individuals with type 1 diabetes, 13 progressed to kidney failure defined by a composite endpoint of progression of albuminuria and incident ESKD [26]. In another study, researchers reported no relationship between LTL and incident ESKD in individuals with type 1 diabetes with impaired kidney function [29]. In our study, rLTL alone had modest performance for identifying incident ESKD in type 2 diabetes but including rLTL led to significant improvement in the ability to stratify ESKD risk. Together with our previous findings, the present study suggests that rLTL may be applied as a biomarker in clinical practice to predict the future risk of diabetic complications, including CVD [5], renal disease and mortality [6, 10]. This may help us to identify individuals at high risk, as candidates for early intensive and personalised treatment to delay the disease progression, with such intervention possibly even facilitating disease regression if the individual is identified early enough and treated aggressively*.*

Several studies have suggested an important role for oxidative stress in shortening of telomeres [31]. In animal studies, telomere dysfunction supressed the expression of peroxisome proliferator-activated receptor γ and coactivator 1α and 1β, leading to impaired mitochondrial function, decreased gluconeogenesis and increased levels of reactive oxygen species [32]. Individuals with diabetic nephropathy had reduced mitochondrial DNA content in the circulation with reduced maximal respiration and reserve capacity compared with individuals without diabetic nephropathy [33]. Based on these observations, the researchers proposed that systemic mitochondrial dysfunction initiated by hyperglycaemia-induced mitochondrial DNA damage might be implicated in the pathogenesis of diabetic nephropathy. Other studies support links between oxidative stress and inflammation in people with kidney dysfunction. Among individuals on dialysis, inflammation, evident by increased plasma C-reactive protein, and duration of dialysis were associated with oxidative stress measured by plasma levels of thiobarbituric acid reactive substances (TBARS) [34]. In another study including 64 individuals with ESKD, acute-phase proteins were positively associated with biomarkers of oxidative stress [35]. In our analysis, the associations between rLTL shortening and ESKD, CKD and eGFR decline were independent of traditional risk factors albeit with some attenuation. Although we did not measure oxidative stress markers, the progressive attenuation of the risk associations with adjustment suggested that an abnormal milieu might contribute towards telomere shortening via inflammation and oxidative stress. The adverse effect of telomere shortening on mitochondrial function might worsen oxidative stress and accelerate kidney dysfunction [32]. Future studies, including those incorporating Mendelian randomisation, may further clarify the relationship between telomere shortening and the development of diabetic complications.

In general, women had longer telomeres than men, and this difference became less evident with onset of menopause [36]. However, individuals with diabetes who had multiple metabolic risk factors were reported to have shorter rLTL than those without diabetes [37]. A suboptimal milieu including diabetes could induce telomere attrition, which might attenuate the effects of sex difference on rLTL. In this analysis, we did not detect sex differences in rLTL, similar to the findings of other studies including Chinese people with diabetes [29, 38–40]. However, we did find a sex difference in the rLTL-associated complications, with a stronger association between rLTL and incident ESKD in women compared with men. Given that men had more risk factors than women, possibly contributing towards their higher risk of cardiovascular and kidney complications, telomere shortening might take on more important prognostic significance in women. This sex difference was also observed in our previous report on the risk association between rLTL and incident CVD [5].

Our study has several strengths. This was a large single-centre prospective cohort where rLTL was measured in more than 5000 Chinese individuals with type 2 diabetes. We have developed a robust laboratory assay to measure rLTL, with excellent CVs and with consistent results when rLTL was analysed with different reference standards. The follow-up period of 14 years was the longest among similar reports and gave our study an adequate number of event rates and robust statistical power. The comprehensive documentation of confounders at baseline together with the capturing of eGFR values during follow-up allowed us to perform analyses using different measures to estimate progression of kidney dysfunction with adjustment for confounders. Our study also has limitations. First, rLTL was measured only at one time point, limiting our ability to explore the impact of the attrition rate of rLTL on the risk of ESKD and the impact of therapeutics on rLTL. The sample size for eGFR decline was small and validation studies are needed to confirm results from this single cohort. We cannot completely exclude the possibility of residual confounding, although we believe our analyses have considered the contribution of most major confounders.

In conclusion, rLTL was independently associated with incident ESKD, CKD, eGFR decline and rapid loss of kidney function in Chinese individuals with type 2 diabetes. We proposed that shorter rLTL might be a useful biomarker for propensity of progression of kidney disease in type 2 diabetes although the mechanism underlying this risk association remains to be elucidated.

## Supplementary information

ESM 1 (PDF 252 kb)

## Data Availability

The datasets generated during and/or analysed during the current study are available from the corresponding author on reasonable request.

## Abbreviations

ACR: Albumin/creatinine ratio
AKI: Acute kidney injury
CKD: Chronic kidney disease
ESKD: End-stage kidney disease
HBG: Human β-globin
HKDR: Hong Kong Diabetes Register
LTL: Leucocyte telomere length
NRI: Net reclassification improvement
NTC: No-template control
PWH: Prince of Wales Hospital
QC: Quality control
rLTL: Relative leucocyte telomere length
rLTL_QC: Relative leucocyte telomere length calculated by the reference QC sample
ROC: Receiver operating characteristic
