# Supplementary material for: Relative leucocyte telomere length is associated with incident end-stage kidney disease and rapid decline of kidney function in type 2 diabetes: analysis from the Hong Kong Diabetes Register
Source: Diabetologia. 2021 Nov 22;65(2):375–86. doi: 10.1007/s00125-021-05613-1 (PMC8741666; doi:10.1007/s00125-021-05613-1)
Supplement: Supplementary file 1 — (PDF 252 kb) [file 125_2021_5613_MOESM1_ESM.pdf]

ESM Table 1. Baseline characteristics according to the tertiles of relative leucocyte telomere length.

Baseline variables	T1≤4.20	4.20<T2≤5.13	T3>5.13	*P value*
Number	1362	1361	1362	
Age, year	56.02 ± 12.48	53.90 ± 12.91	52.94 ± 12.24	**<0.001**
Male, %	631 (46.3%)	612 (45.0%)	610 (44.8%)	0.419
Duration of diabetes, year	6.71 ± 6.14	5.99 ± 6.12	5.44 ± 5.95	**<0.001**
Current smoker, %	190 (14.0%)	209 (15.4%)	198 (14.6%)	0.653
Ever smoked, %	433 (31.8%)	388 (28.6%)	375 (27.6%)	**0.016**
Current drinker, %	125 (9.2%)	123 (9.1%)	165 (12.2%)	**0.011**
Ever drinker, %	303 (22.4%)	268 (19.8%)	289 (21.4%)	0.510
SBP, mmHg	134.83 ± 20.17	131.51 ± 18.92	130.59 ± 18.68	**<0.001**
DBP, mmHg	76.81 ± 11.00	75.63 ± 10.52	75.44 ± 10.06	**0.001**
BMI, kg/m^2	25.13 ± 3.89	25.36 ± 4.16	25.36 ± 4.25	0.139
HbA$_{1c}$, mmol	61.69 ± 20.35	59.57 ± 19.74	57.80 ± 18.89	**<0.001**
HbA$_{1c}$, %	7.79 ± 1.86	7.60 ± 1.81	7.44 ± 1.73	**<0.001**
FPG, mmol/l	8.97 ± 3.31	8.65 ± 3.31	8.32 ± 3.23	**<0.001**
TC, mmol/l	5.28 ± 1.11	5.09 ± 1.13	5.08 ± 1.10	**<0.001**
HDL-C, mmol/l	1.31 ± 0.38	1.33 ± 0.38	1.36 ± 0.40	**0.001**
LDL-C, mmol/l	3.24 ± 0.99	3.02 ± 0.92	2.98 ± 0.93	**<0.001**
Triacylglycerol, mmol/l	1.33 [0.91, 2.02]	1.30 [0.90, 1.90]	1.35 [0.95, 1.97]	0.956*
Urinary ACR, mg/mmol	1.94 [0.83, 8.15]	1.49 [0.65, 5.24]	1.37 [0.68, 4.25]	**<0.001***
eGFR, ml min^{-1} 1.73 m^{-2}	89.66 ± 17.00	90.78 ± 17.02	91.77 ± 16.46	**0.001**
RBC, $10^{\wedge 12}$/l	4.71 ± 0.57	4.71 ± 0.56	4.77 ± 0.59	**0.030**
HB, g/l	13.94 ± 1.50	13.91 ± 1.51	13.98 ± 1.50	0.513
WBC, $10^{\wedge 9}$/l	7.35 ± 4.07	7.17 ± 1.86	7.08 ± 1.88	**0.021**
Diagnosed comorbidity				
Retinopathy, %	343 (25.2%)	303 (22.3%)	295 (21.7%)	**0.029**
Neuropathy, %	293 (21.5%)	232 (17.0%)	157 (11.5%)	**<0.001**
Microalbuminuria, %	387 (29.2%)	320 (24.4%)	311 (23.7%)	**0.001**
Macroalbuminuria, %	165 (12.5%)	126 (9.6%)	97 (7.4%)	**<0.001**
Use of medications				
Lipid lowering drugs, %	171 (12.6%)	213 (15.7%)	245 (18.0%)	**<0.001**
Antihypertensive drugs, %	539 (39.6%)	523 (38.4%)	514 (37.7%)	0.325
Oral antihyperglycemic drugs, %	875 (64.2%)	925 (68.0%)	952 (69.9%)	**0.002**
Insulin use, %	191 (14.0%)	180 (13.2%)	164 (12.0%)	0.125
RAS inhibitors (ACEIs or ARBs), %	244 (17.9%)	220 (16.2%)	236 (17.3%)	0.684

All data are shown as mean ± SD, median (Q1-Q3) or proportion in %; All comparisons of clinical characteristics was conducted by using linear regression for continuous variables and logistic regression for category variables with/without adjustment for age; *logarithmic

transformation was used in TG and ACR. A one-unit increase in log (TG) or log (ACR+1) corresponds to a 10-fold increase in TG, or (ACR+1), respectively.

Abbreviations: BMI, body mass index; SBP, systolic blood pressure; DBP, diastolic blood pressure; HbA_{1c}, Haemoglobin A1c; FPG, fasting plasma glucose; TC, total cholesterol; TG, triacylglycerol; HDL-C, high-density lipoprotein cholesterol; LDL-C, low-density lipoprotein cholesterol; eGFR, estimated glomerular filtration rate; ACR, urinary albumin to creatinine ratio; RAS inhibitors, renin angiotensin system inhibitors; ACEIs, angiotensin converting enzyme inhibitors; ARBs, angiotensin receptor blockers. T1, tertile 1; T2, tertile 2, T3, tertile 3.

ESM Table 2. Cox regression analysis of the association between shorter rLTL and incident end stage kidney disease in subgroups

ESKD	Events/Total (%)	Hazard Ratio (95% CI)		
		Unadjusted model	Fully adjusted model	*p value*[*]
Overall	564/4085 (13.8%)	1.21 (1.13-1.30)	1.11 (1.03-1.19)	**0.007**
Males	263/1853 (14.2%)	1.18 (1.07-1.31)	1.07 (0.95-1.21)	0.240
Females	301/2232 (13.5%)	1.24 (1.13-1.36)	1.11 (1.01-1.22)	**0.025**
Age at diagnosis of diabetes				
< 40 years	107/1017 (10.5%)	1.19 (1.01-1.40)	1.24 (1.03-1.49)	**0.026**
40+ years	457/3067 (14.9%)	1.20 (1.11-1.29)	1.09 (1.00-1.18)	**0.039**

rLTL, relative leucocyte telomere length calculated by negative control (water); ESKD, end stage kidney disease. * P value for fully adjusted model; Full adjusted model included age, sex, duration of diabetes, BMI, SBP, DBP, HbA_{1c}, LDL-C, Log (TG), HDL-C, eGFR, Log (ACR), retinopathy, neuropathy and CVD at baseline. Sex and retinopathy were included as strata variables.

ESM Table 3 Competing Risk Regression analysis showing subdistribution HRs of rLTL for incident end stage kidney disease.

Subdistribution HRs	rLTL (each unit decrease) sHR	*p value*
Model 1	1.19 (1.11-1.26)	**<0.001**
Model 2	1.14 (1.07-1.22)	**<0.001**
Model 3	1.12 (1.06-1.19)	**<0.001**
Model 4	1.10 (1.02-1.18)	**0.009**
Model 5	1.10 (1.02-1.18)	**0.009**

sHR, subdistribution hazards ratio; rLTL, relative leucocyte telomere length calculated by negative control (water).

Sex and retinopathy were included as strata variables.

Model 1: without adjustment

Model 2: adjusted for age and sex

Model 3: Model 2 + adjusted for duration of diabetes, BMI, SBP and DBP

Model 4: Model 3 + adjusted for HbA1c, LDL-C, $\log_{10}$ (triacylglycerol), HDL-C, eGFR and $\log_{10}$ (ACR)

Model 5: Model 4 + retinopathy, neuropathy and CVD at baseline.

ESM Table 4 Hazard ratio (95% CI) for incident end stage kidney disease according to tertiles (T) of rLTL

Variables	Model 1		Model 2		Model 3		Model 4		Model 5	
	HR (95%CI)	*p value*	HR (95%CI)	*p value*	HR (95%CI)	*p value*	HR (95%CI)	*p value*	HR (95%CI)	*p value*
T1 (rLTL≤4.20)	1.77 (1.43-2.19)	**<0.001**	1.56 (1.26-1.93)	**<0.001**	1.48 (1.19-1.83)	**<0.001**	1.41 (1.12-1.78)	**0.003**	1.39 (1.10-1.75)	**0.006**
T2 (4.20<rLTL≤5.13)	1.28 (1.02-1.60)	**0.032**	1.22 (0.97-1.53)	0.085	1.19 (0.95-1.50)	0.127	1.19 (0.93-1.51)	0.169	1.17 (0.92-1.49)	0.210
T3 (rLTL>5.13)					Reference					
Age, years			1.04 (1.03-1.04)	**<0.001**	1.02 (1.01-1.03)	**<0.001**	1.00 (0.99-1.02)	0.526	1.01 (0.99-1.02)	0.349
Diabetes duration, years					1.04 (1.03-1.05)	**<0.001**	1.01 (1.00-1.02)	0.169	1.00 (0.99-1.02)	0.883
BMI, kg/m^2					1.02 (0.99-1.04)	0.194	0.99 (0.97-1.01)	0.368	0.99 (0.97-1.02)	0.508
SBP, mmHg					1.02 (1.02-1.03)	**<0.001**	1.01 (1.01-1.02)	**<0.001**	1.01 (1.01-1.02)	**<0.001**
DBP, mmHg					0.98 (0.97-0.99)	**0.001**	0.98 (0.97-0.99)	**<0.001**	0.98 (0.97-0.99)	**<0.001**
HbA1c, mmol/mol							1.01 (1.01-1.02)	**<0.001**	1.01 (1.01-1.02)	**<0.001**
LDL-C, mmol/l							0.86 (0.78-0.94)	**0.001**	0.85 (0.77-0.93)	**0.001**
Log (TG)							1.39 (0.90-2.16)	0.142	1.49 (0.96-2.32)	0.075
HDL-C, mmol/l							1.22 (0.95-1.56)	0.113	1.24 (0.97-1.57)	0.088
eGFR, ml min^{-1} 1.73 m^{-2}							0.98 (0.97-0.98)	**<0.001**	0.98 (0.97-0.98)	**<0.001**
Log (ACR)							3.20 (2.80-3.65)	**<0.001**	2.92 (2.54-3.36)	**<0.001**
Neuropathy									1.18 (0.96-1.45)	0.119
CVD at baseline									0.88 (0.67-1.16)	0.355

HR, hazard ratio; BMI, body mass index; SBP, systolic blood pressure; DBP, diastolic blood pressure; HbA_{1c}, Haemoglobin A1c; LDL-C, low-density lipoprotein cholesterol; TG, triacylglycerol; HDL-C, high-density lipoprotein cholesterol; eGFR, estimated glomerular filtration rate; ACR, urinary albumin to creatinine ratio; rLTL, relative leucocyte telomere length calculated by negative control (water)

Sex and retinopathy were included as strata variables.

Model 1: without adjustment

Model 2: adjusted for age and sex

Model 3: Model 2 + adjusted for duration of diabetes, BMI, SBP and DBP

Model 4: Model 3 + adjusted for HbA1c, LDL-C, $\log_{10}$ (triacylglycerol), HDL-C, eGFR and $\log_{10}$ (ACR)

Model 5: Model 4 + retinopathy, neuropathy and CVD at baseline.

ESM Table 5 Linear regression analysis of the association between rLTL and eGFR change during follow-up period.

Variables	Model 1		Model 2		Model 3		Model 4		Model 5	
	Beta ± SE	*p value*	Beta ± SE	*p value*	Beta ± SE	*p value*	Beta ± SE	*p value*	Beta ± SE	*p value*
Intercept	-5.25 ± 0.32	**<0.001**	-1.28 ± 0.49	**0.009**	2.01 ± 0.86	**0.019**	0.65 ± 1.31	0.620	0.62 ± 1.30	0.634
rLTL, ΔΔCt	0.42 ± 0.07	**<0.001**	0.35 ± 0.07	**<0.001**	0.27 ± 0.06	**<0.001**	0.14 ± 0.06	**0.021**	0.13 ± 0.06	**0.029**
Age, years			-0.06 ± 0.01	**<0.001**	-0.02 ± 0.01	**0.006**	-0.01 ± 0.01	0.121	-0.01 ± 0.01	0.146
Male			-0.37 ± 0.16	**0.019**	-0.60 ± 0.16	**<0.001**	-0.52 ± 0.15	**0.001**	-0.48 ± 0.15	**0.002**
Diabetes duration, years					-0.11 ± 0.01	**<0.001**	-0.04 ± 0.01	**0.003**	-0.02 ± 0.01	0.144
BMI, kg/m^2					0.00 ± 0.02	0.836	0.03 ± 0.02	0.071	0.03 ± 0.02	0.116
SBP, mmHg					-0.05 ± 0.01	**<0.001**	-0.03 ± 0.01	**<0.001**	-0.03 ± 0.01	**<0.001**
DBP, mmHg					0.03 ± 0.01	**0.006**	0.04 ± 0.01	**<0.001**	0.04 ± 0.01	**<0.001**
HbA1c, mmol/mol							-0.030 ± 0.004	**<0.001**	-0.029 ± 0.004	**<0.001**
LDL-C, mmol/l							-0.04 ± 0.08	0.655	-0.03 ± 0.08	0.710
Log (TG)							-0.40 ± 0.36	0.269	-0.56 ± 0.36	0.119
HDL-C, mmol/l							0.03 ± 0.21	0.873	0.01 ± 0.21	0.980
eGFR, ml min^{-1} 1.73 m^{-2}							0.01 ± 0.01	**0.019**	0.01 ± 0.01	**0.050**
Log (ACR)							-3.86 ± 0.15	**<0.001**	-3.67 ± 0.15	**<0.001**
Retinopathy									-1.07 ± 0.18	**<0.001**
Neuropathy									-0.65 ± 0.20	**0.001**
CVD at baseline									0.11 ± 0.24	0.655

BMI, body mass index; SBP, systolic blood pressure; DBP, diastolic blood pressure; HbA_{1c}, Haemoglobin A1c; LDL-C, low-density lipoprotein cholesterol; TG, triacylglycerol; HDL-C, high-density lipoprotein cholesterol; eGFR, estimated glomerular filtration rate; ACR, urinary albumin to creatinine ratio; rLTL, relative leucocyte telomere length calculated by negative control (water)

Model 1: without adjustment

Model 2: adjusted for age and sex

Model 3: Model 2 + adjusted for duration of diabetes, BMI, SBP and DBP

Model 4: Model 3 + adjusted for HbA1c, LDL-C, log_{10} (triacylglycerol), HDL-C, eGFR and log_{10} (ACR)

Model 5: Model 4 + retinopathy, neuropathy and CVD at baseline.

ESM Table 6 Sensitivity analysis after excluding incident ESKD defined within 30 days of AKI diagnosis.

Variables	Model 1		Model 2		Model 3		Model 4		Model 5	
	HR (95%CI)	*p value*	HR (95%CI)	*p value*	HR (95%CI)	*p value*	HR (95%CI)	*p value*	HR (95%CI)	*p value*
rLTL, ΔΔCt	1.17 (1.08-1.27)	<0.001	1.13 (1.04-1.22)	0.004	1.11 (1.03-1.20)	0.01	1.09 (1.00-1.19)	0.047	1.09 (1.00-1.19)	0.052
Age, years			1.03 (1.03-1.04)	<0.001	1.02 (1.01-1.03)	<0.001	1.00 (0.99-1.02)	0.749	1.00 (0.99-1.01)	0.849
Diabetes duration, years					1.03 (1.02-1.05)	<0.001	1.01 (0.99-1.03)	0.314	1.01 (0.99-1.03)	0.35
BMI, kg/m^2					1.01 (0.99-1.04)	0.348	0.99 (0.96-1.01)	0.336	0.99 (0.96-1.01)	0.338
SBP, mmHg					1.02 (1.01-1.03)	<0.001	1.01 (1.00-1.02)	0.004	1.01 (1.00-1.02)	0.004
DBP, mmHg					0.99 (0.97-1.00)	0.021	0.98 (0.97-0.99)	0.003	0.98 (0.97-0.99)	0.004
HbA1c, mmol/mol							1.01 (1.01-1.02)	<0.001	1.01 (1.01-1.02)	<0.001
LDL-C, mmol/l							0.83 (0.74-0.92)	0.001	0.82 (0.74-0.92)	0.001
Log (TG)							1.43 (0.86-2.39)	0.17	1.46 (0.87-2.45)	0.149
HDL-C, mmol/l							1.17 (0.88-1.55)	0.287	1.18 (0.89-1.56)	0.261
eGFR, ml min^{-1} 1.73 m^{-2}							0.98 (0.97-0.98)	<0.001	0.98 (0.97-0.98)	<0.001
Log (ACR)							3.49 (2.97-4.10)	<0.001	3.42 (2.91-4.03)	<0.001
Neuropathy									1.19 (0.93-1.52)	0.168
CVD at baseline									1.00 (0.73-1.36)	0.977

HR, hazard ratio; BMI, body mass index; SBP, systolic blood pressure; DBP, diastolic blood pressure; HbA_{1c}, Haemoglobin A1c; LDL-C, low-density lipoprotein cholesterol; TG, triacylglycerol; HDL-C, high-density lipoprotein cholesterol; eGFR, estimated glomerular filtration rate; ACR, urinary albumin to creatinine ratio; rLTL, relative leucocyte telomere length calculated by negative control (water). Sex and retinopathy were included as strata variables.

Model 1: without adjustment

Model 2: adjusted for age and sex

Model 3: Model 2 + adjusted for duration of diabetes, BMI, SBP and DBP

Model 4: Model 3 + adjusted for HbA1c, LDL-C, $\log_{10}$ (triacylglycerol), HDL-C, eGFR and $\log_{10}$ (ACR)

Model 5: Model 4 + retinopathy, neuropathy and CVD at baseline.

ESM Table 7 Cox regression showing the association between rLTL calculated based on QC materials and incident end stage kidney disease.

Variables	Model 1		Model 2		Model 3		Model 4		Model 5	
	HR (95%CI)	*p value*	HR (95%CI)	*p value*	HR (95%CI)	*p value*	HR (95%CI)	*p value*	HR (95%CI)	*p value*
rLTL_QC, ΔΔCt	1.24 (1.16-1.34)	**<0.001**	1.17 (1.09-1.26)	**<0.001**	1.15 (1.07-1.24)	**<0.001**	1.13 (1.04-1.23)	**0.003**	1.14 (1.05-1.23)	**0.002**
Age, years			1.04 (1.03-1.04)	**<0.001**	1.02 (1.01-1.03)	**<0.001**	1.01 (1.00-1.02)	0.316	1.01 (0.99-1.02)	0.360
Diabetes duration, years					1.02 (1.01-1.04)	**0.001**	1.00 (0.99-1.02)	0.784	1.00 (0.99-1.02)	0.853
BMI, kg/m^2					1.02 (0.99-1.04)	0.179	0.99 (0.97-1.01)	0.427	0.99 (0.97-1.01)	0.435
SBP, mmHg					1.02 (1.01-1.03)	**<0.001**	1.01 (1.01-1.02)	**<0.001**	1.01 (1.01-1.02)	**<0.001**
DBP, mmHg					0.98 (0.97-0.99)	**0.001**	0.98 (0.97-0.99)	**<0.001**	0.98 (0.97-0.99)	**<0.001**
HbA1c, mmol/mol							1.01 (1.01-1.02)	**<0.001**	1.01 (1.01-1.02)	**<0.001**
LDL-C, mmol/l							0.85 (0.78-0.94)	**0.001**	0.85 (0.77-0.93)	**0.001**
Log (TG)							1.43 (0.92-2.21)	0.108	1.48 (0.95-2.30)	0.080
HDL-C, mmol/l							1.22 (0.96-1.55)	0.109	1.23 (0.96-1.56)	0.096
eGFR, ml min^{-1} 1.73 m^{-2}							0.98 (0.97-0.98)	**<0.001**	0.98 (0.97-0.98)	**<0.001**
Log (ACR)							2.99 (2.60-3.44)	**<0.001**	2.94 (2.55-3.38)	**<0.001**
Neuropathy									1.20 (0.98-1.48)	0.078
CVD at baseline									0.88 (0.67-1.15)	0.337

HR, hazard ratio; BMI, body mass index; SBP, systolic blood pressure; DBP, diastolic blood pressure; HbA_{1c}, Haemoglobin A1c; LDL-C, low-density lipoprotein cholesterol; TG, triacylglycerol; HDL-C, high-density lipoprotein cholesterol; eGFR, estimated glomerular filtration rate; ACR, urinary albumin to creatinine ratio; rLTL, relative leucocyte telomere length calculated by QC.

Sex and retinopathy were included as strata variables.

Model 1: without adjustment

Model 2: adjusted for age and sex

Model 3: Model 2 + adjusted for duration of diabetes, BMI, SBP and DBP

Model 4: Model 3 + adjusted for HbA1c, LDL-C, $\log_{10}$ (triacylglycerol), HDL-C, eGFR and $\log_{10}$ (ACR)

Model 5: Model 4 + retinopathy, neuropathy and CVD at baseline.

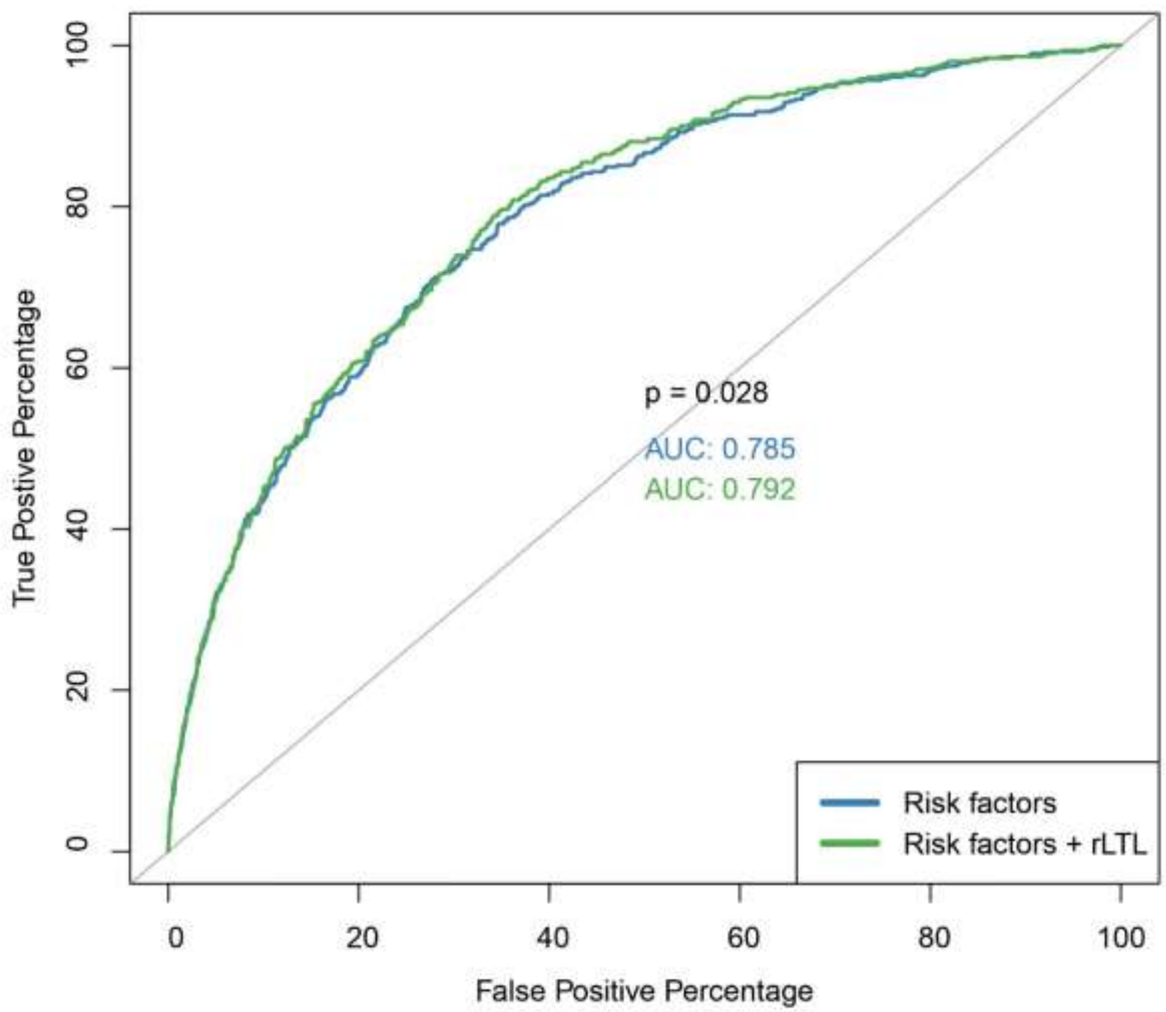

ESM Figure 1. Receiver operating characteristic curve (ROC) for prediction of end stage kidney disease. Traditional risk factors models with or without inclusion of rLTL (Area under the curve: 0.792 (0.772-0.813) vs. 0.785 (0.764-0.806), P=0.002). rLTL: relative leucocyte telomere length calculated by negative control (water).

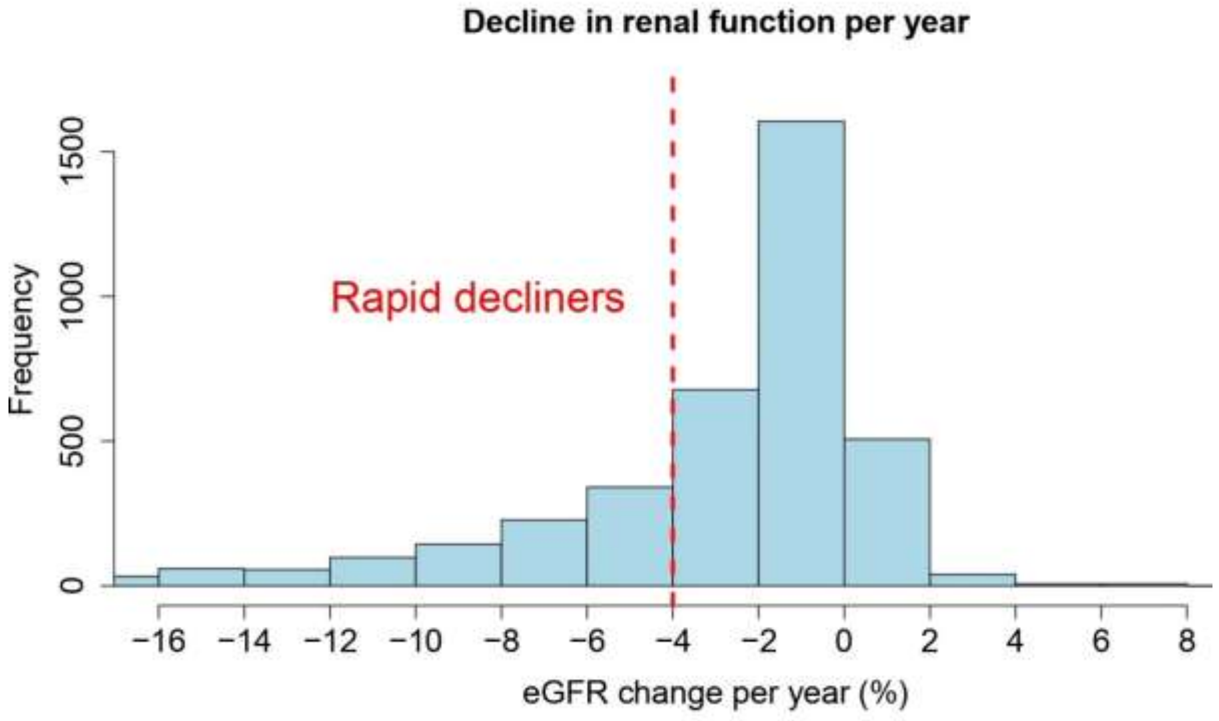

ESM Figure 2. The histogram of decline in renal function per year during follow-up. Patients with eGFR decline of >4%/year were defined as rapid decliners.

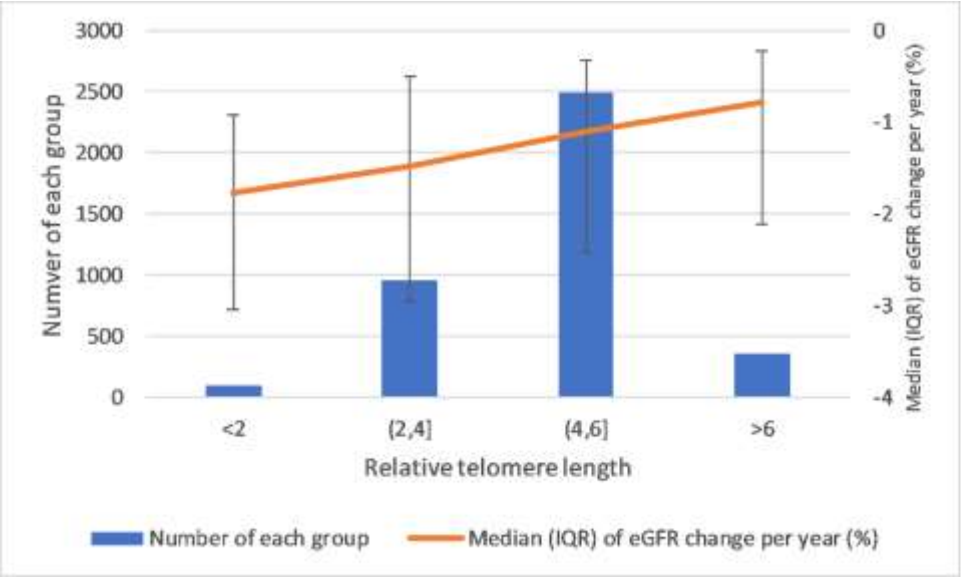

ESM Figure 3. Association between rLTL at baseline and median (IQR) of eGFR change per year during the follow-up period.

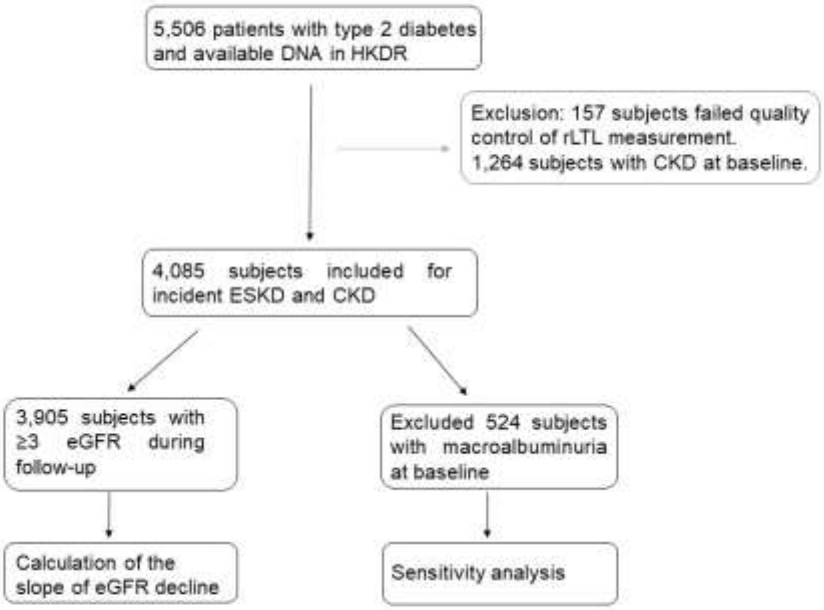

ESM Figure 4. Flow diagram of patient selection

Abbreviation: HKDR, Hong Kong Diabetes Register; rLTL, relative leucocyte telomere length; CKD, chronic kidney disease; ESKD, end stage kidney disease; eGFR, estimated glomerular filtration rate
